# Gene Promoter Methylation in Endometrial Carcinogenesis

**DOI:** 10.1007/s12253-018-0489-2

**Published:** 2018-11-14

**Authors:** Karlijn M. C. Cornel, Kim Wouters, Koen K. Van de Vijver, Anneke A. M. van der Wurff, Manon van Engeland, Roy F. P. M. Kruitwagen, Johanna M. A. Pijnenborg

**Affiliations:** 1 GROW- School for Oncology &Developmental Biology, Maastricht University Medical Centre, Maastricht, Netherlands; 2 Department of Obstetrics and Gynaecology, Maastricht University Medical Centre, P. Debyelaan 25, 6229 HX Maastricht, The Netherlands; 3 Department of Pathology, Maastricht University Medical Centre, Maastricht, Netherlands; 4 Department of Pathology, Ghent University Hospital, Ghent, Belgium; 5 Department of Pathology, Elisabeth-TweeSteden, Tilburg, The Netherlands; 6 Department of Obstetrics and Gynaecology, Radboud University Medical Centre, Nijmegen, The Netherlands

**Keywords:** Methylation, Endometrial hyperplasia, Endometrial cancer, P16, K-Ras, hMLH1

## Abstract

Up to 60% of untreated atypical hyperplastic endometrium will develop into endometrial carcinoma (EC), and for those who underwent a hysterectomy a coexisting EC is found in up to 50%. Gene promoter methylation might be related to the EC development. The aim of this study is to determine changes in gene promoter profiles in normal endometrium, atypical hyperplasia (AH) and EC in relation to *K-Ras* mutations. A retrospective study was conducted in patients diagnosed with endometrial hyperplasia with and without subsequent EC. Promoter methylation of *APC, hMLh1, O6-MGMT, P14, P16, RASSF1, RUNX3* was analysed on pre-operative biopsies, and correlated to the final histological diagnosis, and related to the presence of *K-Ras* mutations. In the study cohort (n=98), differences in promoter methylation were observed for *hMLH1*, *O6-MGMT*, and *P16.* Promoter methylation of *hMLH1* and *O6-MGMT* gradually increased from histologically normal endometrium to AH to EC; 27.3, 36.4% and 38.0% for *hMLH1 and 8.3%, 18.2%* and 31.4% for *O6-MGMT*, respectively. *P16* promoter methylation was significantly different in AH (7.7%) compared to EC (38%). *K-Ras* mutations were observed in 12.1% of AH, and in 19.6% of EC cases. No association of *K-Ras* mutation with promoter methylation of any of the tested genes was found. In conclusion, *hMLH1* and *O6-MGMT* promoter methylation are frequently present in AH, and thus considered to be early events in the carcinogenesis of EC, whereas *P16* promoter methylation was mainly present in EC, and not in precursor lesions supporting a late event in the carcinogenesis.

**Electronic supplementary material:**

The online version of this article (10.1007/s12253-018-0489-2) contains supplementary material, which is available to authorized users.

## Introduction

Endometrial cancer (EC) is the most common malignancy of the female genital tract in Europe and North America. In the Netherlands every year approximately 1,900 women are diagnosed with EC with a mortality rate of approxmately 400 women [1]. The incidence of EC has increased markedly during the last decades, due to both an increased life-expectancy and obesity [2]. The so-called type I tumours (*i.e.* typically low grade endometrioid subtype) are thought to be induced by unopposed estrogen stimulation of the endometrium resulting in hyperplasia with or without atypia, with subsequently progression into EC [3, 4]. The risk of endometrial hyperplasia and progression to cancer is related to the presence of cellular atypia. In 2014 the World health organisation described a new classification system to distinguish endometrial hyperplasia, abandoning the previously used distinction between simple and complex hyperplasia and focusing solely on the presence or absence of atypia [5]. The risk of developing EC when endometrial hyperplasia without atypia is present is around 3%, and increases up to 60% in atypical endometrial hyperplasia (AH). Epigenetic studies explain this risk difference by the observation of no epigenetic changes in hyperplasia without atypia, compared to multiple epigenetic changes in hyperplasia with atypia [4–12].

It is noteworthy that in up to 50% of performed hysterectomies for AH a coexistent EC is found [6, 10, 12–14]. Although the majority of these ECs are diagnosed at an early stage, low histological grade and low stage of disease, Giede et al described a 16% chance of high risk EC, histological grade 2, and advanced stage disease, in patients pre-operatively diagnosed with only AH [14]. The presence of a coexistent carcinoma may alter the surgical treatment approach. In addition, 20% of patients with these type I tumours, that develop out of endometrial hyperplasia, with, a presumed good outcome, present with recurrent disease [8, 9, 14]. Understanding of the progression from AH to EC might contribute to improved selection for hormonal treatment for fertility preservation for young patients, as well as for patients that are not suitable for surgery due to comorbidity.

Gene promoter methylation is frequently present in endometrial carcinoma, mainly in type I EC. Promoter methylation of *hMLH1*, *APC* and *RASSF1A* are considered to be early events in endometrial carcinogenesis [8, 9, 15–21]. Arafa et al. demonstrated that even in normal endometrium, adjacent to the endometrioid adenocarcinoma, a high frequency of *RASSF1A* and *RARb2* promoter methylation was observed [22]. Berg et al. demonstrated that *K-Ras* and *PI3K* activation, loss of *PTEN*, and *PIK3CA* mutations are also early events in the endometrial carcinogenesis and are often present AH lesions. This supports the hypothesis that in AH (epi)genetic mutations are already present, which may result in the progression to endometrial carcinoma [23, 24]. There is evidence that in colorectal cancer and lung carcinoma *O6-MGMT* hypermethylation precedes *K-Ras* mutations [20, 25]. In EC it is shown that *K-Ras* mutations are closely related to micro satellite instability (MSI) [26]. These data, as well as the fact that estrogen is considered to be an epimutagen, support the fact that epigenetic alterations may be an important mechanism in the progression of endometrial hyperplasia into cancer [27].

The purpose of the current study was to determine gene promoter methylation patterns in pre-hysterectomy histological samples of patients with endometrial hyperplasia diagnosed by endometrial sampling prior to hysterectomy, in relation to their final pathological diagnosis. Furthermore, *K-Ras* mutations were analysed and correlated to gene methylation profile.

## Material and Methods

### Patients and Tissue Specimens

All patients diagnosed with endometrial hyperplasia; simple or complex hyperplasia with or without atypia between 1996 and 2011 at the departments of pathology of the Maastricht University Medical Centre and the Elisabeth-Tweesteden hospital Tilburg, who underwent hysterectomy within 9 months after endometrial sampling, were retrieved by the Dutch National Pathology Register (PALGA). All specimens were handled in a coded fashion as prescribed by the Dutch national guidelines for secondary use of specimen (“Human Tissue and Medical Research: Code of conduct for responsible use”). Histology from the endometrium sampling and final histology and diagnosis after hysterectomy were systematically reviewed by one of the two pathologists (KvdV, AvdW) according to the WHO 2014 classification [28, 29]. Histology prior to surgery was obtained by endometrial aspiration, hysteroscopic guided biopsy or dilatation & curettage (D&C). In patients with more than one sample the last sample before surgery was used for analysis.

**Fig. 1:**
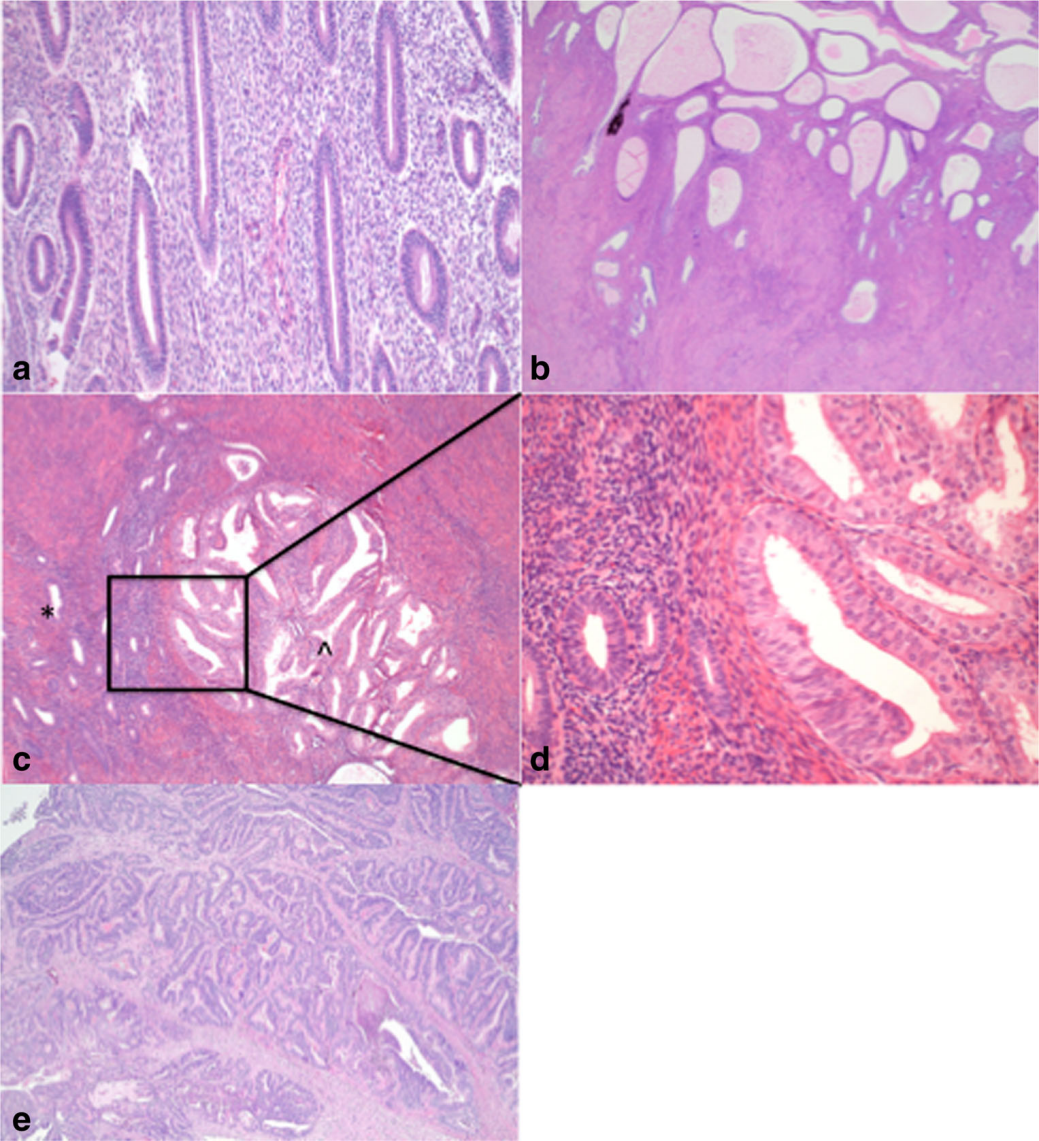
Histology of the endometrium. **a** Normal proliferative endometrium in a pre-menopausal period. **b** Normal endometrium with cystic atrophy (post-menopausal). **c** and **d** Atypical endometrial hyperplasia (^), adjacent to normal endometrium (*). **e** Endometrioid endometrial carcinoma, grade 1

Clinical and pathological characteristics were collected from the patient’s medical charts including: age at diagnosis, Body Mass index (BMI), parity, use of hormonal replacement therapy (HRT), hypertension, diabetes mellitus, menopausal state, smoking, time till hysterectomy, final histopathological diagnosis and FIGO 2009 stage [30]. Final diagnosis was prescribed according to the RCOG Guideline ‘Management of hyperplasia’ [29]; normal endometrium (pre- and postmenopausal), endometrial hyperplasia without atypia, endometrial hyperplasia with atypia (AH) and endometrial carcinoma (EC), as illustrated in Fig. 1. All patients were treated by hysterectomy and bilateral salpingo-oophorectomy. The study was approved by the Medical Ethical Committee of the Maastricht University Medical Center and the Elisabeth-Tweesteden hospital, Tilburg.

### DNA Isolation

After identification of the area of normal endometrium, atypical hyperplasia or EC by the pathologist, tissue was manually dissected from five consecutive 20 μm sections of the paraffin embedded tissue. Genomic DNA was extracted using a proteinase K (Qiagen) digestion followed by DNA isolation using the Puregene DNA Isolation Kit (Gentra Systems).

### Gene Promoter Methylation Analysis

DNA methylation in the CpG island of the genes was determined as described previously [31] and investigated for the following genes; *APC, hMLh1, O6-MGMT, P14, P16, RASSF1, RUNX3*. To enable MSP analysis on DNA retrieved from formalin-fixed, paraffin embedded tissue, a nested MSP was performed as described by van Engeland et al. [32]. All MSP’s were performed with a control for unmethylated alleles (DNA from normal lymphocytes), a control for methylated alleles (in vitro methylated DNA of normal lymphocytes treated with Sssl methyltransferase (New England Biolabs)) and a negative control (H2O). A total of 6 μl of each MSP reaction was loaded on a non-denaturing 6% polyacrylamide gel, stained with ethidium bromide and visualised under UV illumination. The presence of a PCR product in respectively the U and M lane indicates the presence of unmethylated or methylated alleles. Cases methylated in one of two cases were considered as methylated. The primer sets for all tested genes are listed in Supplementary table 1.

### K-Ras Mutation

As a template for *K-Ras* codon 12 and 13 a 179 bp product was amplified and used as a template for the amplification of a 114 bp fragment. 250 ng genomic DNA was added to 50 mmol/l MgCl_2,_ 18.15 μl MQ, 2.50 μl PCR buffer (10x), 0.25 μl deoxynucleoside triphosphate (dNTP) (Pharmacia, Uppsala, Sweden), 0.25 μl of Flank primers F 5′-AGGCCTGCTGAAAATGACTGAATA-3′ and 5′-CTGTATCAAAGAATGGTCCTGCAC-3′, and 0.1 μl platinum- Taq 5 U/l (Invitrogen, Breda, The Netherlands) (annealing temperature 50_C).

The final analyses were performed with 5 μl 100x dilution (1:100 2μl PCR product in 198 ul 0.1xTE), 18.4 μl MQ, 2.5 μl PCR buffer (10x), 0.75 μl 50 mmol/l MgCl_2,_ 0.25 μl dNTP, 0.25 μl of primers 5′-AAAATGACTGAATATAAACTTGTGG-3 and 5′-CTCTATTGTTGGATCATATTCGTC-3′ and 0.1 μl platinum- Tag 5 U/l.

Electrophoresis on 2% agarose gels was used to check the size and amount of the PCR products. Clean up was performed using 2 μl ExoSAP-IT kit (product number 78201) with 5 μl PCR product. Sequencing reaction was performed using the Big Dye sequencing 1.1 kit (1.5 μl Big Dye buffer, 1 μl Big Dye mix, 1.6 μl primer 1 μM (5′ TGTAAAACGACGGCCAGT 3′ and 5′ CAGGAAACAGCTATGACC 3′), 4.9 μl MilliQ added to 1 μl cleaned up PCR product). Afterward the PCR products were separated.

### Primary Outcome

Primary outcome was defined as the differences in gene promoter methylation between normal endometrium, AH and EC in the pre-operative samples.

### Secondary Outcome

Secondary outcome was defined as the correlation of gene promoter methylation with the presence of *K-Ras* mutations and clinicopathological factors.

### Data Analysis

The SPSS software program (22.0) was used for statistical analysis. Median values were calculated for the patient’s age and BMI. To test whether the differences for gene promoter methylation and *K-Ras* mutation between the three patient groups (normal endometrium, AH and EC) were significant, ANOVA with post HOC analyses were performed.

All tests of statistical significance were two-sided, and a p-value of 0.05 was considered significant.

## Results

### Patient Characteristics

A total of 99 patients met the inclusion criteria. Patients were classified in three different groups based on the final histological examination of the hysterectomy specimen categorized as normal endometrium, AH and EC (Fig. 1). One patient with simple hyperplasia without atypia was excluded to optimize the homozygosity of our study cohort resulting in 98 suitable for analysis. Patient characteristics according to final pathology are shown in Table 1.

**Table 1:** Patient characteristics according to final pathological diagnosis

	Normal endometrium (n=14)	Atypical endometrial hyperplasia (n=33)	Endometrial carcinoma (n=51)	P value
Age at time of hysterectomy (mean)in years	57 (40-76)	64 (41-93)	63 (35-84)	p<0.05*
BMI kg/m2 (median)	27.2 (19.0-37.0)	30.2 (21.0-44.1)	30.2 (19.0-43.0)	n.s.
Tumor grade				n.a.
Grade I			39	
Grade II			4	
Grade III			0	
Unknown			8	
FIGO stage (2009)				n.a.
IA			33	
IB			10	
II			2	
IIIA			1	
IIIB			0	
IIIC			0	
IV			0	
Unkown			5	

Table 1 * Significant difference between normal endometrium and atypical endometrial hyperplasia p=0.041,. n.s. = not statistically significant. n.a. not applicable

The mean age of the study cohort was 62 years (35-93) with a mean BMI of 30.9 kg/m^2^ (19.0-43.0). The majority (79%) of patients were postmenopausal at time of diagnosis.

There was a only statistical significant difference in age at the time of hysterectomy between the patients with normal endometrium compared to patients with atypical endometrial hyperplasia.

Table 2 shows the correlation between the pre-operative histological diagnosis and the final post-operative histological diagnosis. The agreement between the pre- and post-operative histological diagnosis was 55%. In 35% of cases there was an upgrade of the pre-operative histological diagnosis and in 10% there was a downgrade of the pre-operative diagnosis (Table 3).

**Table 2:** Pre-operative histological diagnosis compared to final histological diagnosis

Pre-operative histological diagnosis	Post-operative histological diagnosis	N
Normal endometrium (NE) (N=17)	NE	2
	AH	1
	EC	14
Atypical endometrial hyperplasia (AH) (N=72)	NE	9
	AH	44
	EC	19
Endometrial carcinoma (EC) (N=9)	NE	
	AH	1
	EC	8

Table 2 Nl = normal endometrium, hyp = atypical hyperplastic endometrium, EC = endometrial cancer

**Table 3:** Individual relation between K-Ras mutation and 06-MGMT, P16 and hMlh1 gene promoter methylation

Final pathological diagnosis	Patients with K-Ras mutation	06-MGMT methylation	P16 methylation	hMlh1 methylation
Atypical hyperplastic endometrium	1	yes	no	no
	2	no	unkown	no
	3	yes	yes	no
	4	yes	unknown	no
Endometrial carcinoma	5	no	yes	no
	6	no	yes	yes
	7	no	no	no
	8	no	no	no
	9	no	unknown	yes
	10	no	no	no
	11	no	no	no
	12	yes	yes	no
	13	yes	no	yes
	14	yes	no	yes

### Gene Promoter Methylation

**Fig. 2:**
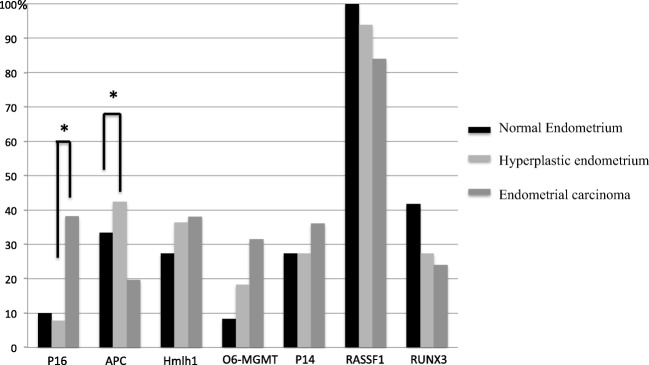
Percentage of gene promoter methylation according to final pathology. Gene promoter methylation in percentage according to final histological classification (normal endometrium, atypical endometrial hyperplasia and grade 1 endometrioid endometrial carcinoma). * = p <0.05

Results of the percentages of gene promoter methylation for the tested genes are illustrated in Fig. 2, according the endometrial tissue: normal endometrium, atypical hyperplastic endometrium, endometrial carcinoma. For *hMLH1* (n=96) a non-statistically significant increase in promoter methylation was seen in the development from normal endometrium to AH into EC:*,* 27,3% (n=3), 36.4% (n=12) and 38.0% (n=19). A comparable pattern was seen for *O6-MGMT* (n=96) with promoter methylation in 8.3% (n=1), 18.2 % (n=6) and 31.4% (n=16) respectively. There was a significant increase in promoter methylation for *P16* (n=70) in patients with EC 38.2 % (n=13) compared to AH 7.7% (n=2) (p<0.05). The difference in methylation between normal endometrium (10% n=1) compared to AH is not significantly different. The *APC* (n= 96) promoter methylation was more frequently present in patients with AH 42.4 % (n=14) compared to the patients with EC 19.6% (n=10) and was significantly different (p<0.05). No significantly difference was seen for normal endometrium (33.3% n=4) compared to AH and/or EC.

For all other tested genes: p14 (n=94), RASSF1 (n=94), RUNX3 (n=95) no pattern in gene promoter methylation between normal endometrium, atypical endometrial hyperplasia and EC was seen.

### K-Ras Mutations

*K-Ras* analysed was performed in a total of 99 cases. In none of the patients with the diagnosis normal endometrium (n=14) *K-Ras* mutations were found, whereas *K-Ras* mutations were found in AH and EC in respectively 12.1% (4/33) and 19.6% (10/51). The difference was not statistically significant.

**Fig. 3:**
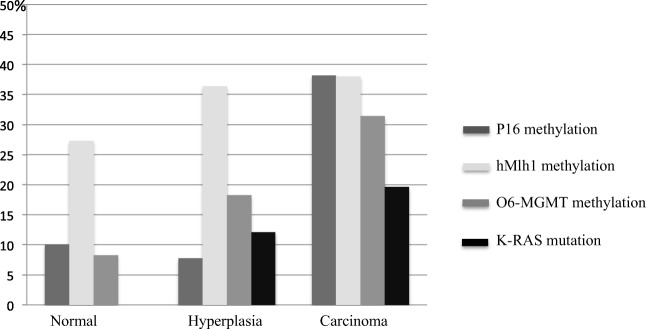
Promoter methylation for P16, hMLH1, and 06-MGMT, and K-Ras mutation according to final pathological classification

In Fig. 3 the results of *K-Ras* mutations and promoter methylation for *hMLH1*, *O6-MGMT*, and *P16* are summarized. BMI, age and tumour grade were not significantly related with the promoter methylation of any of the tested genes.

Data of the *K-Ras* mutations in relation to *hMLH1, O6-MGMT* and *P16* promoter methylation for both AH and EC are presented in Supplementary Table 2.

## Discussion

In the current study we have demonstrated that promoter methylation of *hMLH1* and *06-MGMT* was frequently seen in premalignant endometrial tissue, whereas *P16* promoter methylation was more frequently observed in EC. Also *K-Ras* mutations were present in AH, yet these mutations were not related to any of the promoter methylated genes.

Although gene promoter methylation has frequently been described in EC, data in precursor lesions are limited.

The *hMLH1* is one of the genes involved in the DNA mismatch repair mechanism and related to microsatellite instability (MSI) in several cancers. We observed *hMLH1* promoter methylation in 38% of EC cases, which is in accordance with previous studies [7, 8, 33]. Additionally, in AH we found promoter methylation in 36.4%, and even in 25% of normal endometrial tissue *hMLH1* promoter methylation was found, in contrast to previous studies that did not identify promoter methylation in normal endometrium [7, 8, 34].

Esteller et al. showed a relation between *hMLH1* promoter methylation and MSI. In their study they showed 7 out of 21 AH cases positive for *hMLH1* promoter methylation, (33.3%) in line with our data. Furthermore, 40% of EC showed *hMLH1* promoter methylation and in 91% of the cases MSI was seen. Guida et al. showed that especially the combination of *hMLH1* promoter methylation and *P16* promoter methylation is more frequently seen in endometrial hyperplasia and endometrial carcinoma compared to normal endometrium [14, 15, 34].

The presence of MSI caused by mismatch repair deficiency either through gene promoter methylation or gene mutation seems clinically relevant. A strong relation between the expression of the programmed cell death 1 protein (PD-1) and programmed cell death 1 ligand 1 (PD-L1) as well as tumour-infiltrating lymphocytes (CD8+) and MSI was recently demonstrated [35, 36]. This might indicate that immune checkpoint inhibitors (anti-PD-1/PD-L1 antibody) could be effective in endometrial cancers with MSI. The presence of MSI may be a biomarker for good response to PD-1/PD-L1 immunotherapy in endometrial cancer [35, 36].

*O6-MGMT* is known to play an important role in the carcinogenesis for different type of tumours. In normal cells it’s involved in DNA damage repair and prevent mismatch errors during DNA transcription and replication. Increased gene promoter methylation and thereby silencing of this gene may result in progression to the development of neoplasms. In the present study we found a trend of increased promoter methylation for the *O6-MGMT* from normal endometrium (8.3%) into endometrial carcinoma (31.4%), which is in accordance with two other studies [12, 15]. In contrast, Rimel et al. did not found any *O6-MGMT* promoter methylation in 120 endometrial cancer tissues and 6 endometrial cancer cell lines [37, 38].

In our study we observed a significant increase of *P16* promoter methylation from AH to EC, which supports the hypothesis that *P16* promoter methylation might contribute to the development of EC. The tumour suppressor *P16* acts as a tumour suppressor that inhibits the cyclin-dependent kinase 2A, resulting in cell cycle arrest, and consequently, promoter methylation of *P16* consequently results in cell growth and proliferation. In our study cohort *P16* promoter methylation was found to increase from normal endometrium to EC. Previous studies showed conflicting results of the *P16* promoter methylation in normal endometrium. Most studies show no gene promoter methylation at all [39–41]. Banno et al. did not observe any *P16* promoter methylation in his series of 92 cases including normal, premalignant and malignant endometrial tissue [8, 15].

Salvesen et al. showed *P16* promoter methylation in one out of 138 EC cases, however most of these cases were high grade EC, which are assumed to be more frequently related to *p53* mutations than to epigenetic factors [40]. In addition, low grade EC development is known to be specifically related to precursor lesions such as atypical hyperplasia and is known to be related to intracellular estrogen levels, whereas for high grade EC the carcinogenesis is different [42]. The same results are seen by Yanokura et al., who investigated 17 EC mostly high grade ECs and did not find any promoter methylation in those cases [41]. Others have shown *P16* promoter methylation in up to 75% [15, 33, 34, 39–41]. Hu et al. performed a meta-analysis including 6 studies and showed an Odds ratio of 13.5 (97% CI 5.5-33.3) for the relation between *P16* promoter methylation and EC, although the type of EC was not specified in this series. These data suggest that *P16* promoter methylation is late event in the carcinogenesis of endometrioid EC and therefore interesting with respect to its low presence in AH and patients prognosis.

Current data support the existing data that *hMLH1* and *O6-MGMT* promoter methylation are early events in de progression from normal endometrium into endometrial cancer. Whereas *P16* promoter methylation is showed to be a late event in EC development.

We found an increased rate of *K-Ras* mutation for AH compared to endometrial cancer cases. No *K-Ras* mutations were found in patients with a final diagnosis of normal endometrium. *K-*Ras mutations are frequently seen in combination with microsatellite instability (MSI) [43–45]. Van der Putten et al. studied the presence of *K-*Ras mutations in EC and the adjacent endometrial tissue and found increased mutation rates in EC with adjacent hyperplastic endometrium, but not in EC with adjacent atrophic endometrium. This suggests a role for *K-Ras* mutation in the development of endometrioid EC.

The strength of this study is the homogenous groups of AH cases and low-grade endometrioid ECs. Furthermore our group of controls consists of mostly postmenopausal women and, because the increased incidence of endometrial cancer in the post-menopausal period, this is of important value when discussing the development of endometrial cancer.

Limitations of the study are the relatively small sample size, the control group not being matched to the cases in regarding to age and BMI, although the latter did not differ between groups.

### Clinical Implications

Epigenetic alterations in precursor lesions of EC may be clinically relevant to estimate the risk of a coexisting carcinoma, as well as contribute to the response of hormonal treatment for those women who would like to preserve fertility, and those who have an increased risk for surgery.

## Conclusion

*hMLH1* and *O6-MGMT* promoter methylation and *K-Ras* mutation seem to be early events in the carcinogenesis of EC and are frequently present in atypical endometrial hyperplasia. Whereas *P16* promoter methylation is mainly present in EC, and not in precursor lesions supporting as a late event in the EC carcinogenesis. These data support the importance of epigenetic changes in de development of EC.

## Electronic Supplementary Material

ESM 1 (DOCX 30.4 kb)

## Abbreviations

APC: APC Adenomatous polyposis coli
hMLH1: Human mutL homolog 1
O6-MGMT: O-6-methylguanine-DNA methyltransferase
P14: Cyclin-dependent kinase inhibitor 2A (alternative reading frame)
P16: Cyclin-dependent kinase inhibitor 2A
RASSF1: Ras association (RalGDS/AF-6) domain family member 1
RUNX3: Runt-related transcription factor 3
K-Ras: proto-oncogene
