# Supplementary material for: Gene Promoter Methylation in Endometrial Carcinogenesis
Source: Pathol Oncol Res. 2018 Nov 14;25(2):659–67. doi: 10.1007/s12253-018-0489-2 (PMC6449282; doi:10.1007/s12253-018-0489-2)
Supplement: Supplementary file 1 — (DOCX 30.4 kb) [file 12253_2018_489_MOESM1_ESM.docx]

Supplementary table 1. Primers used for gene promoter methylation analysis.

| GENES | PRIMERS |  |
| --- | --- | --- |
| APC | flank up | TGG GYG GGG TTT TGT GTT TTA TT |
|  | flank down | TAC RCC CAC ACC CAA CCA ATC |
|  | us | GTG TTT TAT TGT GGA GTG TGG GTT |
|  | uas | CCA ATC AAC AAA CTC CCA ACA A |
|  | ms | TAT TGC GGA GTG CGG GTC |
|  | mas | TCG ACG AAC TCC CGA CGA |
| Hmlh1 | flank up | TTT TGA YGT AGA YGT TTT ATT AGG GT |
|  | flank down | AAA ACR ATA AAA CCC TAT ACC TAA TCT ATC |
|  | us | TGT GTG TTT GTT GTT TGT TAT ATA TTG TTT |
|  | uas | ACC ACC TCA TCA TAA CTA CCC ACA |
|  | ms | GTT CGT CGT TCG TTA TAT ATC GTT C |
|  | mas | CCT CAT CGT AAC TAC CCG CG |
| 06-MGMT | flank up | GYG TTT YGG ATA TGT TGG GAT AGT T |
|  | flank down | AAA CTC CRC ACT CTT CCR AAA AC |
|  | us | TTT GTG TTT TGA TGT TTG TAG GTT TTT GT |
|  | uas | AAC TCC ACA CTC TTC CAA AAA CAA AAC A |
|  | ms | TTT CGA CGT TCG TAG GTT TTC GC |
|  | mas | GCA CTC TTC CGA AAA CGA AAC G |
| P16 | flank up | GGG TTG GTT GGT TAT TAG AGG GT |
|  | flank down | RAC CRT AAC CAA CCA ATC AAC C |
|  | us | GTT GGT TAT TAG AGG GTG GGG TGG ATT GT |
|  | uas | AAC CAA AAA CTC CAT ACT ACT CCC CAC CA |
|  | ms | TTA TTA GAG GGT GGG GCG GAT CGC |
|  | mas | GAA AAC TCC ATA CTA CTC CCC GCC G |
| RASSF1A | flank up | GTT TAG TTT GGA TTT TGG GGG AG |
|  | flank down | CCC RCA ACT CAA TAA ACT CAA ACT C |
|  | us | GGG GTT TGT TTT GTG GTT TTG TTT |
|  | uas | AAC ATA ACC CAA TTA AAC CCA TAC TTC A |
|  | ms | GGG TTC GTT TTG TGG TTT CGT TC |
|  | mas | TAA CCC GAT TAA ACC CGT ACT TCG |
| RUNX3 | flank up | TAG TGG GGA TGG GAG GTG TT |
|  | flank down | CCC CAA AAC CCA AAT AAA A |
|  | us | GGA TGG GAG GTG TTT GAG ATG TT |
|  | uas | CTA CAA AAC ACA TCC AAA ACA AAA CA |
|  | ms | ATG GGA GGT GTT CGA GAC GTC |
|  | mas | AAC GCA TCC AAA ACG AAA CG |

Supplementary table 2. Individual relation between K-Ras mutation and 06-MGMT, P16 and hMlh1 gene promoter methylation

| **Final pathological diagnosis** | **Patients with K-Ras mutation** | **06-MGMT methylation** | **P16 methylation** | **hMlh1 methylation** |
| --- | --- | --- | --- | --- |
| Atypical hyperplastic endometrium | yes | no | yes | no |
|  | yes | no | yes | yes |
|  | yes | no | no | no |
|  | yes | no | yes | unknown |
|  | no | no | no | yes |
|  | no | no | no | no |
|  | no | yes | no | no |
|  | no | no | no | no |
|  | no | no | no | no |
|  | no | no | no | no |
|  | no | no | no | no |
|  | no | no | no | no |
|  | no | yes | no | no |
|  | no | yes | no | unknown |
|  | no | yes | no | no |
|  | no | yes | no | unknown |
|  | no | no | no | no |
|  | no | yes | no | no |
|  | no | yes | no | no |
|  | no | no | no | no |
|  | no | yes | no | no |
|  | no | no | no | unknown |
|  | no | no | no | no |
|  | no | no | no | no |
|  | no | no | yes | no |
|  | no | yes | no | no |
|  | no | yes | yes | unknown |
|  | no | no | no | unknown |
|  | no | yes | no | no |
|  | no | no | yes | no |
|  | no | no | no | no |
|  | no | no | no | unknown |
|  | unknown | yes | no | no |
| Endometrial carcinoma | yes | yes | yes | no |
|  | yes | no | yes | yes |
|  | yes | yes | no | yes |
|  | yes | no | no | no |
|  | yes | no | no | yes |
|  | yes | no | no | no |
|  | yes | no | no | unknown |
|  | yes | yes | no | no |
|  | yes | yes | no | no |
|  | yes | no | yes | no |
|  | no | yes | yes | yes |
|  | no | yes | yes | yes |
|  | no | no | no | no |
|  | no | yes | no | yes |
|  | no | no | yes | yes |
|  | no | yes | yes | yes |
|  | no | yes | no | yes |
|  | no | no | yes | yes |
|  | no | no | yes | yes |
|  | no | no | yes | yes |
|  | no | no | no | no |
|  | no | ? | no | no |
|  | no | no | no | unknown |
|  | no | no | no | no |
|  | no | no | no | no |
|  | no | yes | no | no |
|  | no | yes | no | no |
|  | no | no | no | no |
|  | no | yes | yes | no |
|  | no | no | yes | no |
|  | no | no | no | unknown |
|  | no | no | no | unknown |
|  | no | no | no | unknown |
|  | no | no | no | unknown |
|  | no | yes | no | no |
|  | no | no | no | no |
|  | no | no | no | unknown |
|  | no | no | no | unknown |
|  | no | no | no | unknown |
|  | no | no | no | no |
|  | no | yes | yes | unknown |
|  | no | yes | no | unknown |
|  | no | no | no | unknown |
|  | no | yes | no | unknown |
|  | no | yes | yes | unknown |
|  | no | no | yes | unknown |
|  | no | no | no | no |
|  | no | no | no | unknown |
|  | no | unknown | no | unknown |
|  | unknown | yes | no | no |
|  | unknown | yes | yes | yes |
